# Effectiveness of using a single-shade universal resin composite before internal bleaching: a 15-month follow-up case report

**DOI:** 10.1038/s41415-025-8917-7

**Published:** 2025-11-14

**Authors:** Ryota Takaoka, Zhihao Zhai, Shion Morioka, Kenichi Nomura, Ami Aihara, Masahiro Nishimura

**Affiliations:** 109549317224304922994 Assistant Professor, Department of Fixed Prosthodontics and Orofacial Function, Osaka University Graduate School of Dentistry, Osaka, Japan; 613995705017762060112 Clinical Fellow, Department of Fixed Prosthodontics and Orofacial Function, Osaka University Graduate School of Dentistry, Osaka, Japan; 879186437221912280857 Graduate Student, Department of Fixed Prosthodontics and Orofacial Function, Osaka University Graduate School of Dentistry, Osaka, Japan; 653686957104614247113 Professor, Department of Fixed Prosthodontics and Orofacial Function, Osaka University Graduate School of Dentistry, Osaka, Japan

## Abstract

Single-shade universal resin composites allows light transmission from the surrounding tooth structure and light emission at a specific frequency, and it can reduce skill complexity, chair time, and inventory requirements. The present article aimed to demonstrate the aesthetic outcomes of restoring a tooth with a single-shade universal resin composite before internal bleaching.

A 55-year-old female patient presented with an internally discoloured maxillary left lateral incisor and underwent treatment involving the application of a single-shade resin composite before internal bleaching. Fifteen months after bleaching, the colour of the direct restoration remained in harmony with the surrounding tooth structure, achieving clinically acceptable aesthetics. Direct restoration using a single-shade resin composite before internal bleaching reduced chairside time, preserved tooth structure, and eliminated the need for interim restoration.

The combination of single-shade resin composite restoration and internal bleaching offers a minimally invasive, time-saving, and easy-to-handle treatment option with acceptable aesthetic outcomes.

## Key points

- Using a single-shade universal resin composite before internal bleaching achieves acceptable colour match.
- The combination of single-shade resin composite restoration and internal bleaching offers a minimally invasive, time-saving, and easy-to-handle treatment option.
- Further research is required regarding a single-shade universal resin composite.

## Introduction

To re-establish the missing tooth structure, it is necessary to restore form, function and aesthetics. For all restoration methods, an imperceptible match between the colour of restorative materials and the surrounding tooth structure is essential. Direct restoration methods using resin composites have gained popularity in recent years due to their immediate aesthetic presentation, minimal invasiveness, and low cost.^1^ Unlike indirect restoration, where shade selection can be performed later in the fabrication stage, direct restoration using resin composites requires accurate selection of composite shades and layering of these materials.^2^^,^^3^^,^^4^^,^^5^ This process is skill-sensitive, time-consuming and requires a corresponding inventory.

Single-shade universal resin composites have been introduced to address these shortcomings, leveraging their superior ‘blending effect' or ‘chameleon effect'. These composites can be used with a single universal shade because of their high translucency, which allows light transmission from the surrounding tooth structure and light emission at a specific frequency.^6^ It is well-recognised that this type of composite possesses colour adaptiveness similar to traditional multiple-shade systems.^7^^,^^8^ Thus, using these composites can reduce skill complexity, chair time and inventory requirements.

The internal bleach or walking bleach technique is a well-established approach for enhancing the aesthetics of discoloured non-vital teeth.^9^ Oxidising agents, such as hydrogen peroxide and sodium perborate, are introduced into the pulp chamber after proper root canal obturation. The agent is changed several times until the desired shade is achieved. Due to the shade change, confirmation of tooth shade stabilisation before definitive restoration is strongly recommended.^9^ This leads to the use of interim restorations and prevents achieving aesthetic outcomes at an early stage. However, since single-shade universal resin composites have strong colour-adapting ability, it remains unclear whether this precaution is necessary with these materials.

As most one-shade universal composite resins have a higher tonal compatibility with teeth with a brighter shade, such as B1 and A1, than with teeth with the discoloured teeth, such as C3 and D3,^7^ we hypothesised that one-shade universal composite resins would be compatible with a whitening treatment that which gradually increases the value of the teeth. This article describes a case of achieving aesthetic outcomes by restoring a tooth with a single-shade universal resin composite before internal bleaching.

## Case presentation

**Fig. 1:**
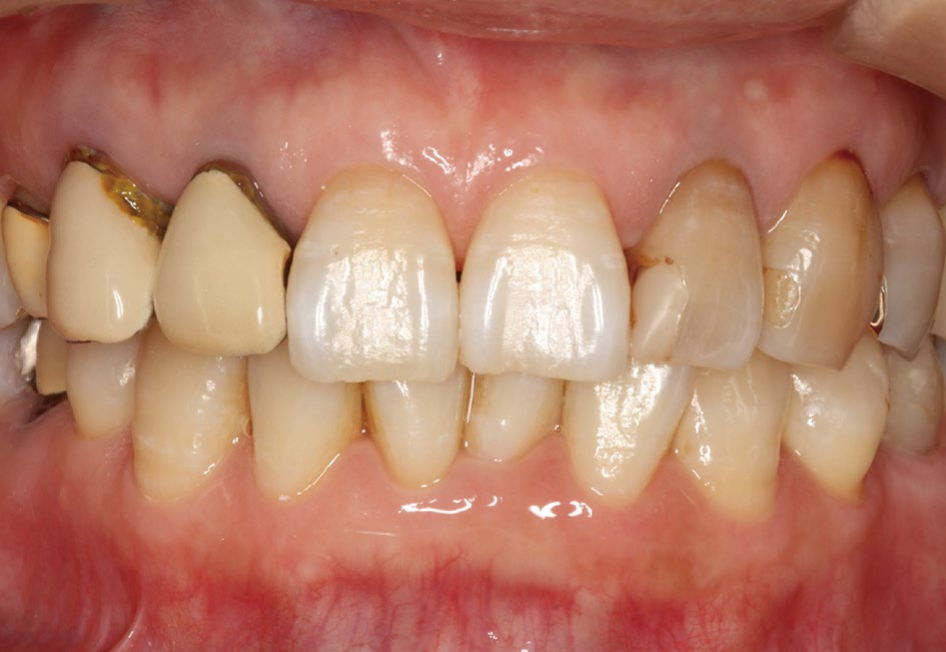
Intra-oral photograph before treatment

A 55-year-old female patient presented with a chief complaint of poor aesthetics in the anterior region (Fig. 1). Pre-treatment intra-oral inspection and photographs (facial and intra-oral) showed that the dental midline was appropriate in the maxillary arch but shifted approximately 1 mm to the left in the mandibular arch. The maxillary anterior gingival margin was considered appropriate. Although the incisal edges of the maxillary central incisors were uneven, no treatment was performed due to the minimal intervention concept and the patient's preference. The remaining incisal edges and occlusal planes were aligned with the lip line. Splinted porcelain-fused-to-metal crowns (PFMs) from the maxillary right lateral incisor to the right first premolar had an unaesthetic form.

Additionally, the shade of PFMs did not match that of the proximal teeth. Secondary caries, likely resulting from marginal misfits, were present around the crown margins. The resin composite restorations of the maxillary left lateral incisor and canine showed secondary caries at the restorative margins. The two teeth were internally discoloured and presented a darker shade than that of the central incisors. Diagnostic radiographs indicated periapical radiolucency of the maxillary right lateral incisor, right canine, right first premolar, left lateral incisor, and left canine. All mentioned teeth, except for the central incisors, had previously undergone root canal treatment. A significant misfit was observed at the prosthetic margin of the maxillary right canine. The coronal margin of the remaining tooth structure was located subgingivally over the entire circumference. Informed consent for extraction of the central incisors was obtained from the patient.

A replacement of the crowns from the maxillary right lateral incisor to the right first premolar was planned. For the maxillary left lateral incisor, restoration renewal using resin composite was planned. Crown restoration was planned for the maxillary left canine because of significant tooth discoloration, large-scale loss of tooth structure, and the need to reconstruct the cuspid-protected articulation.

The PFMs and corresponding post cores were removed. Before root canal treatment, orthodontic extrusion and surgical crown lengthening of the maxillary right canine were performed, achieving an appropriate dentin ferrule. Root canal retreatment was then performed for all teeth from the maxillary right first molar to the left canine, except for the central incisors. After confirming the stabilisation of the gingival margin, splinted porcelain-fused-to-zirconia (PFZ) crowns were fabricated for the maxillary right lateral incisor, canine, and first premolar.

**Fig. 2:**
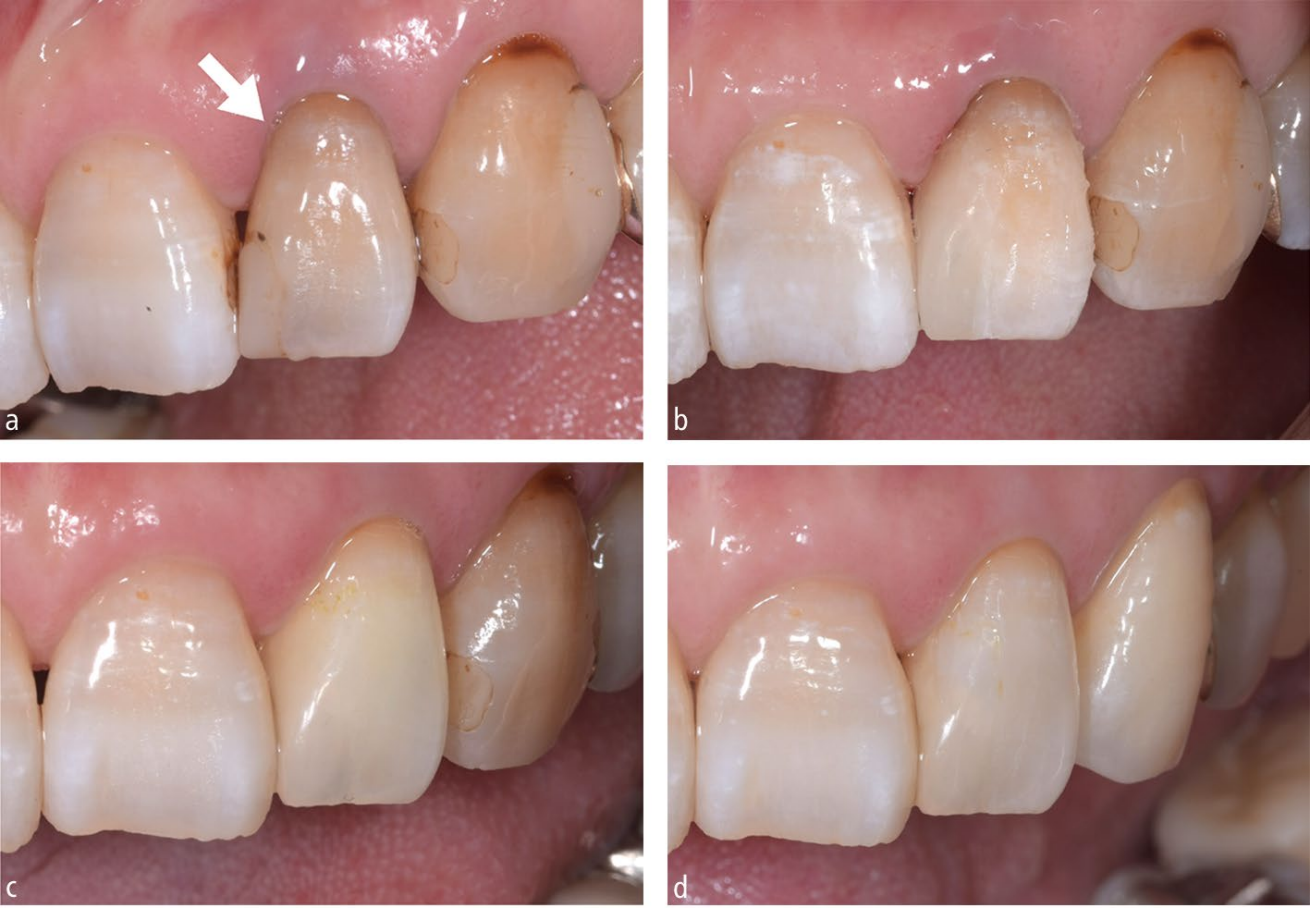
Internal bleaching for the maxillary left lateral incisor. a) Before bleaching. b, c) Tooth shade after the first and second change of bleaching agent. d) After treatment. Note that the single-shade universal resin composite on the mesial adjacent surfaces was not modified after the initial filling

Direct restoration using a single-shade resin composite (Omnichroma, Tokuyama Dental, Japan) was performed on the maxillary left lateral incisor after the root canal treatment. The composite was placed using a sectional matrix (Hawe Adapt, Kerr Dental, USA) and a two-step self-etching bonding agent (CLEARFIL Megabond 2, Kuraray Noritake Dental, Japan). Subsequently, a sodium perborate bleaching agent was applied to the pulp chamber to induce internal bleaching (Fig. 2). The bleaching agent was changed and the tooth shade was reevaluated over three subsequent visits. Temporary sealing using a resin composite (GRACEFIL Flo, GC, Japan) and assessment for leakage were performed at each visit. After confirming with the patient that the target shade was achieved, the bleaching agent was completely removed.

**Fig. 3:**
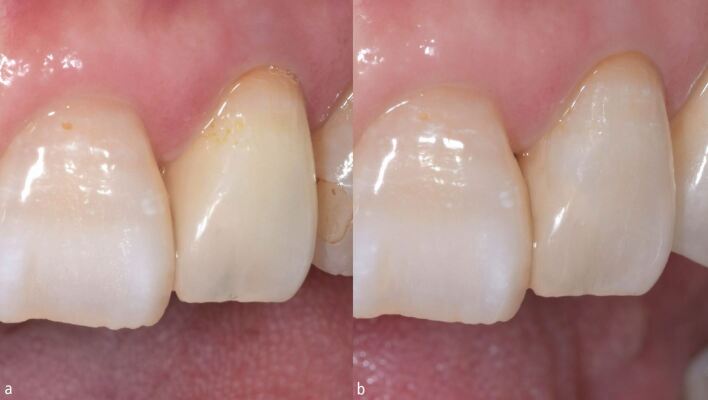
Change in tooth shade of the maxillary lateral incisor after pulp chamber filling with two different composites. a) Gradia Direct AO2. b) SOLIDEX AC2 and Gradia Direct E3

To mimic the hue of dentin, an opaque dentin-shade resin composite (AO2 shade; Gradia Direct, GC, Japan) was applied to the pulp chamber (Fig. 3A). However, the hue of the tooth mismatched with that of the adjacent teeth. Thus, the composite was replaced with a combination of two composites: a cervical shade (AC2 shade, SOLIDEX, Shofu, Japan) for the apical part and an enamel shade (E3 shade, Gradia Direct, GC, Japan) for the coronal part of the pulp chamber (Fig. 3B). The colour of the composite restoration remained consistent with that of the surrounding tooth structure after internal bleaching. A single PFZ crown was fabricated for the maxillary left canine. Due to the internal discoloration, a tooth-coloured die was fabricated for accurate shade presentation.

**Fig. 4:**
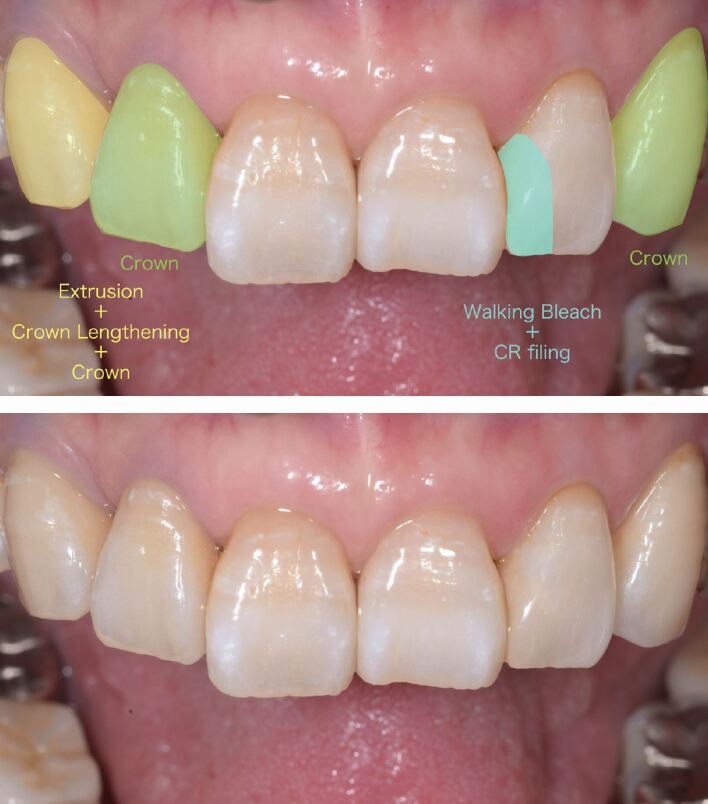
Intra-oral photograph 15 months after direct restoration using a single-shade resin composite

Clinically acceptable aesthetics were achieved in the maxillary anterior region, and the patient was satisfied with the treatment outcome. The aesthetics of the maxillary left lateral incisor were maintained 15 months after direct restoration using a single-shade resin composite (Fig. 4). Written informed consent was obtained from the patient for their personal details to be included and potentially published in the paper.

## Discussion

Herein, we discussed the case where aesthetic outcomes were achieved by restoring a tooth with a single-shade universal resin composite before internal bleaching. The final colour of direct resin composite restorations is influenced by factors such as shade, translucency, and thickness of the composite, as well as the shade of the surrounding tooth structure.^2^ The colour-matching of single-shade resin composites has been well-documented.^7^^,^^8^ However, the effect of internal bleaching on the final colour presentation of these restorations remains unclear. In treatments using multi-shade composites, definitive restorations are usually performed after completing all restorations of the adjacent teeth and bleaching procedures. This implies that interim restorations with non-harmonious shades must be applied throughout the treatment, leading to additional loss of healthy tooth structure from the final restorations and increased chair time. Owing to the blending effect of single-shade resin composites, colour changes in both adjacent and restored teeth may be accommodated. In the presented clinical case, restoration colour harmonisation with the surrounding tooth structure was successfully maintained following internal bleaching and crown restoration of an adjacent tooth.

Internal bleaching is usually performed for teeth with discoloration from pulpal blood degradation.^10^ In addition to its well-proven whitening ability, this technique is minimally invasive and cost-effective compared to veneers and crowns. As demonstrated in this clinical case, when combined with direct restoration using single-shade resin composites, the advantages of these two treatments can be maximally exploited. However, to the best of our knowledge, the efficacy of this combined technique has not yet been evaluated *in vivo* or *in vitro.*

The colour of dentin can significantly influence the final shade of teeth directly restored with resin composites.^11^ In the current clinical case, the tooth presented with a yellowish hue after the initial pulp chamber restoration. After the second restoration, an orangish hue was obtained (Fig. 3). The pulp chamber composite restoration needs a revision in this step until the appropriate hue, brightness, and tone is achieved.

Restoration of Class IV cavities is challenging in terms of colour-match using single-shade resin composites due to the absence of surrounding tooth structures.^12^ While a clinically acceptable colour match was achieved in this case, caution should be exercised when restoring such cavities. According to the manufacturer, the composite shade presented in the present case consisted of filtered light from the material and reflected light from the surrounding tooth structure.^6^ The absence of surrounding tooth surface as a reflection source may lead to a poor colour match. Unfortunately, literature on this subject is limited.

In this case, if the expected bleaching effect had not been achieved using internal bleaching, a veneer or crown would have been applied to the lateral incisor. Fortunately, a sufficient bleaching effect was achieved in this case, and the patient was very satisfied with the results. However, it is always preferable to bleach a tooth before attempting to change its shade with a direct or indirect restoration.

The bleaching effect of carbamide peroxide, hydrogen peroxide and sodium perborate is significant on discoloured, root canal-treated teeth.^9^ In the present case, sodium perborate was used for the bleaching agent. However, Lee reported that carbamide peroxide might be safer because it has very low levels of extraradicular diffusion of hydrogen peroxide in the presence of cemental defects.^13^ In addition, sodium perborate with 10% or 35% carbamide peroxide could be more effective than sodium perborate with distilled water.^14^ Thus, further consideration is required regarding the selection of bleaching agents.

## Summary

The combination of internal bleaching and direct restoration using single-shade resin composites offers a minimally invasive, time-saving, and easy-to-handle treatment option with acceptable aesthetic outcomes. However, further *in vivo* and *in vitro* studies are required to verify the colour adaptation following internal bleaching and the colour presentation in Class IV restorations using such composites.
